# Population mortality before and during the COVID-19 epidemic in two Sudanese settings: a key informant study

**DOI:** 10.1186/s12889-023-17298-9

**Published:** 2024-03-05

**Authors:** Rahaf AbuKoura, Francesco Checchi, Omama Abdalla, Omnia Ibrahim, Ahmed Tom Hemeadan, Ahmed Ali Ahmed Eldirdiri, Direeg Ismail Mohamed, Aljaile Ahmed, Abd Elhameed Ahmed, Nada Abdelmagid, Pasquale Pepe, Maysoon Dahab

**Affiliations:** 1 London School of Hygiene and Tropical Medicine, Department of Infectious Disease Epidemiology, Faculty of Epidemiology and Population Health, London, UK; 2 Y-Peer, Khartoum, Sudan; 3 Ocular Technology Group - International, London, UK; 4 Sudan COVID-19 Research Group, Khartoum, Sudan

**Keywords:** Sudan, Mortality, Capture-recapture, Humanitarian, SARS-CoV-2, COVID-19, Key informant, Multiple systems estimation, Co-production, Community

## Abstract

**Background:**

Population mortality is an important metric that sums information from different public health risk factors into a single indicator of health. However, the impact of COVID-19 on population mortality in low-income and crisis-affected countries like Sudan remains difficult to measure. Using a community-led approach, we estimated excess mortality during the COVID-19 epidemic in two Sudanese communities.

**Methods:**

Three sets of key informants in two study locations, identified by community-based research teams, were administered a standardised questionnaire to list all known decedents from January 2017 to February 2021. Based on key variables, we linked the records before analysing the data using a capture-recapture statistical technique that models the overlap among lists to estimate the true number of deaths.

**Results:**

We estimated that deaths per day were 5.5 times higher between March 2020 and February 2021 compared to the pre-pandemic period in East Gezira, while in El Obeid City, the rate was 1.6 times higher.

**Conclusion:**

This study suggests that using a community-led capture-recapture methodology to measure excess mortality is a feasible approach in Sudan and similar settings. Deploying similar community-led estimation methodologies should be considered wherever crises and weak health infrastructure prevent an accurate and timely real-time understanding of epidemics’ mortality impact in real-time.

**Supplementary Information:**

The online version contains supplementary material available at 10.1186/s12889-023-17298-9.

## Background

The impact of COVID-19, both direct and indirect, on population mortality is difficult to measure in resource-limited and conflict-affected contexts where hospital mortality surveillance and vital registration systems are weak or entirely non-existent [1]. In Sudan, this has meant a lack of timely and accurate data on the mortality impact of the epidemic [2], which in turn limited the understanding of its severity across the country as well as the effectiveness of the response and how to adjust it over time and place.

As of May 2022, the country had recorded 2,967 COVID-19 attributable deaths since the first reported case in March 2020 and through four distinct waves of the epidemic [3]. However, a study in Khartoum estimates that direct COVID-19 deaths were severely under-reported, with up to 98% of COVID-19 deaths being completely missed from official reporting between April and September 2020 [4]. This considerable discordance is in line with findings from Yemen and Syria [5, 6]. Another study conducted in Omdurman, Sudan, found that approximately 54.6% of the population had detectable antibodies against severe acute respiratory syndrome coronavirus 2 (SARS-CoV-2). Additionally, during the first year of the coronavirus disease pandemic, there was a significant 74% increase in the overall population death rates among individuals aged over 50 years [7].There is an urgent need to support demographic and mortality surveillance systems in Sudan and similar contexts to enable accurate situational awareness. This warrants innovative and community-based approaches for measuring population mortality that can circumvent challenges and help fill the gap until exhaustive vital events registration systems are developed [4]. Community-led mortality estimations could contribute to clarifying population needs and provide evidence to inform efforts by national and local actors [8]. This is especially in contexts where health authorities are undermined by political instability. Moreover, COVID-19 has highlighted the importance of excess mortality trends as a key surveillance signal that, combined with transmission dynamic modelling, may help infer the local state of progression of the epidemic [4, 6, 9]. In this study, we sought to estimate excess mortality during the COVID-19 epidemic in two illustrative Sudanese communities using a community-led approach. Community volunteers led the data collection process and played a significant role in the analysis and real-time utilisation of findings.

## Methods

### Study design and settings

**Fig. 1:**
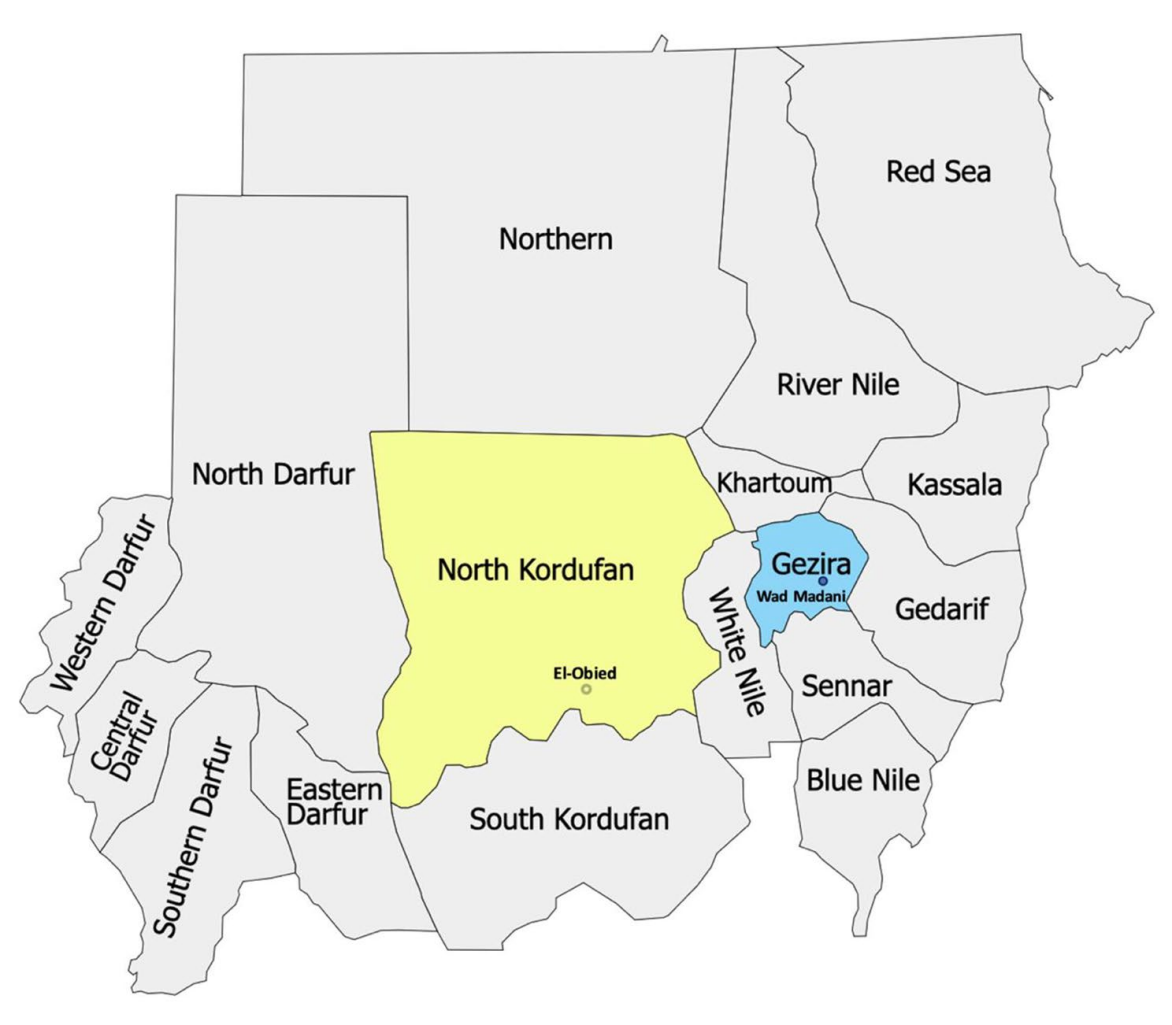
Study Locations

This population-based study applied capture-recapture statistical methods to lists of individual decedents generated by interviewing key community informants, in two distinct locations in Sudan. In North Kurdufan state, we selected Hai al Quba neighbourhood in the capital of the state, El Obeid City, a high-density urban community. In Gezira state, the second most populated state in Sudan, data was collected in Abu Haraz village, a rural community in East Gezira locality located east of Wad Madani, the state’s capital, see Fig. 1. There are no recent or accurate population estimates for the specific study sites available. The choice of the study locations was primarily based on operational criteria, considering factors such as community accessibility and the communities’ inclination and interest to be part of the study.

### Study participants and data collection

The research team in each study site consisted of trained local volunteers, part of the Sudan Youth-Peer-to-Peer Education Network (Y-Peer Sudan). The research teams identified three sets or types of key informants in each study location (see Table 1). They did so using their knowledge of individuals with presumed in-depth knowledge of deaths, either through written information or memory of decedents in their community. Study respondents fulfilled the following criteria: they were part of the identified key informant category, had good knowledge of the study population, and were 18 years old or older. The research teams telephoned the key informants to introduce the study and agree on a convenient date, time, and location for interviews. After obtaining informed consent, the teams collected data from each key informant set based on a standardised questionnaire supported by calendar recall aids via phone calls or in person when deemed more appropriate or safer. The calendar recall aids included memorable dates of major events and holidays to help with respondents’ recall. In the questionnaire, we asked participants to list all known decedents from January 2017 to February 2021. We also asked them to list key information about each decedent, including full name, age, gender, address, and date of death.

**Table 1:** Key informant categories

Study Location	Key informant	Description
Hai al Quba neighbourhood- El Obeid City	Local women’s associations	These have wide geographical distribution and strong relations inside the communities
	Changes and Services Committees	The Changes and Services Committees are popular organisations in localities, villages, markets, and industrial areas that raise citizens’ awareness of their rights and duties. The committees work to provide necessary services, development, security, and stability in their areas.
	Zakat committees	A group of men assigned by the government to distribute the Zakat money to those who need it
Abu Haraz village - East Gezira locality	Community leaders	A community person acts as a focal point between citizens and authorities in Sudan and reports to the Ministry of Interior. Their knowledge of households in the neighbourhood they oversee determines their knowledge of deaths.
	Cemetery caretakers	Cemetery caretakers are responsible for grounds maintenance and burial preparation tasks at a cemetery. They are knowledgeable of any deaths in the community as they are responsible for identifying the best location and excavating the ground to the appropriate size and depth.
	Changes and services committees	As above

### Data Management and analysis

#### Record linkage

We cleaned the data using an iterative process. Using Microsoft Excel [10], we started by manually removing duplicates from each of the three key informant lists. The complete combined list was ordered by name before cross-referencing and linking the records across key informant lists based on name, age, date of death, and address. Identifiers (name, address) were then deleted, and an identification number was assigned to each entry. The numbers 1 and 0 were used to indicate which of the three lists included the death and which did not. When an inconsistency in the recalled deaths details between lists was identified, information about age and date of death was retained from only one of the key informant category lists. This was decided after a discussion with the community-based research teams, who identified the most reliable and credible key informant category. As informants reported a very small number of child deaths and a small number of deaths in 2017, the analysis was restricted to adolescents and adults aged 15 years or older and deaths recalled in 2018 and after.

### *Capture-recapture analysis*

For each site, the capture-recapture analysis examined the overlap among the three informant lists $L$ to estimate the number of decedents who have not been captured by any list. This estimate, when added to the number of decedents appearing on at least one list, provides the total.

Overlap between lists may be represented by eight alternative candidate log-linear Poisson models, each of which features terms for the probability of appearing on any given list, as well as two-way interaction terms representing potential dependencies among lists: these models range from one with no interaction terms to a saturated model featuring interactions${ L}_{1}\times {L}_{2}$, ${L}_{2}\times {L}_{3}$ and ${L}_{1}\times {L}_{3}$. We also included in the models an exposure (the period before and during the COVID-19 pandemic in Sudan). We parameterised models as per Rossi et al. [11]. Adjustment for potential confounding variables (age, gender) did not appreciably affect the point estimates, so we omit these confounders from the final analysis. Each model, once fit, is used to predict ${\widehat{x}}_{000}$, interpretable as each individual’s contribution to ${\widehat{n}}_{000}$, the estimate of uncaptured deaths (i.e., ${\widehat{n}}_{000}=\sum _{i=1}^{N}{\widehat{x}}_{000}$); this quantity was stratified by time period.

Instead of selecting the best-fitting among candidate models, we averaged multiple models using Rossi et al.’s suggested approach [12]. First, we screened out models that did not fit (e.g., due to sparse overlap among lists), yielded an implausible ${\widehat{n}}_{000\left(0\right)}$ (defined as ≥ 10 times the number of listed deaths) or featured a likelihood-ratio test p-value ≥ 0.60 when compared to the saturated model (indicating potential overfitting). For each shortlisted model $i\in \left\{\text{1,2},3\dots K\right\}$, we computed a weight between 0 and 1 ${w}_{i}=\frac{{e}^{-{\varDelta }_{i}/2}}{\sum _{i=1}^{K}{e}^{-{\varDelta }_{i}/2}}$, where ${\varDelta }_{i}={AIC}_{i}-{AIC}_{\text{m}\text{i}\text{n}}$, i.e. the difference between the model’s Akaike Information Criterion (AIC) and the lowest AIC among all shortlisted models. We lastly computed a weighted average estimate ${\widehat{n}}_{000\left(0\right)}=\sum _{i=1}^{K}{w}_{i}{\widehat{n}}_{000\left(0\right),i}$.

### Public involvement and engagement

Through the Y-Peer volunteer research teams, members of the study communities were involved in the design, data collection, analysis, and write-up of this study.

## Results

Data were collected from 16 January 2021 to 20 February 2021. The pre-pandemic period was set to be between 1 January 2018 and 13 March 2020 (the first reported COVID-19 case in Sudan [3]). The period after the start of the pandemic was from 14 March 2020 till the completion of data collection. 51 participants from the identified key informant sets responded with a 12% refusal rate across the study sites.

### Abu Haraz village - East Gezira locality

Overall, the three lists contained records for 174 deaths (12 dated before 2018 and thus excluded), resulting in three lists with unique records for 162 decedents, with moderate overlap (see Additional Fig. 1). Table 2 shows the eight candidate models fitted to the three-list data up to 20 February 2021 and the resulting model averages. Overall, we estimated that 203 (95%CI 41 to 1344) deaths were not captured on any list, yielding a total estimated death toll of 365 (95%CI 203 to 1506) up to 20 February 2021.

**Fig. 2:**
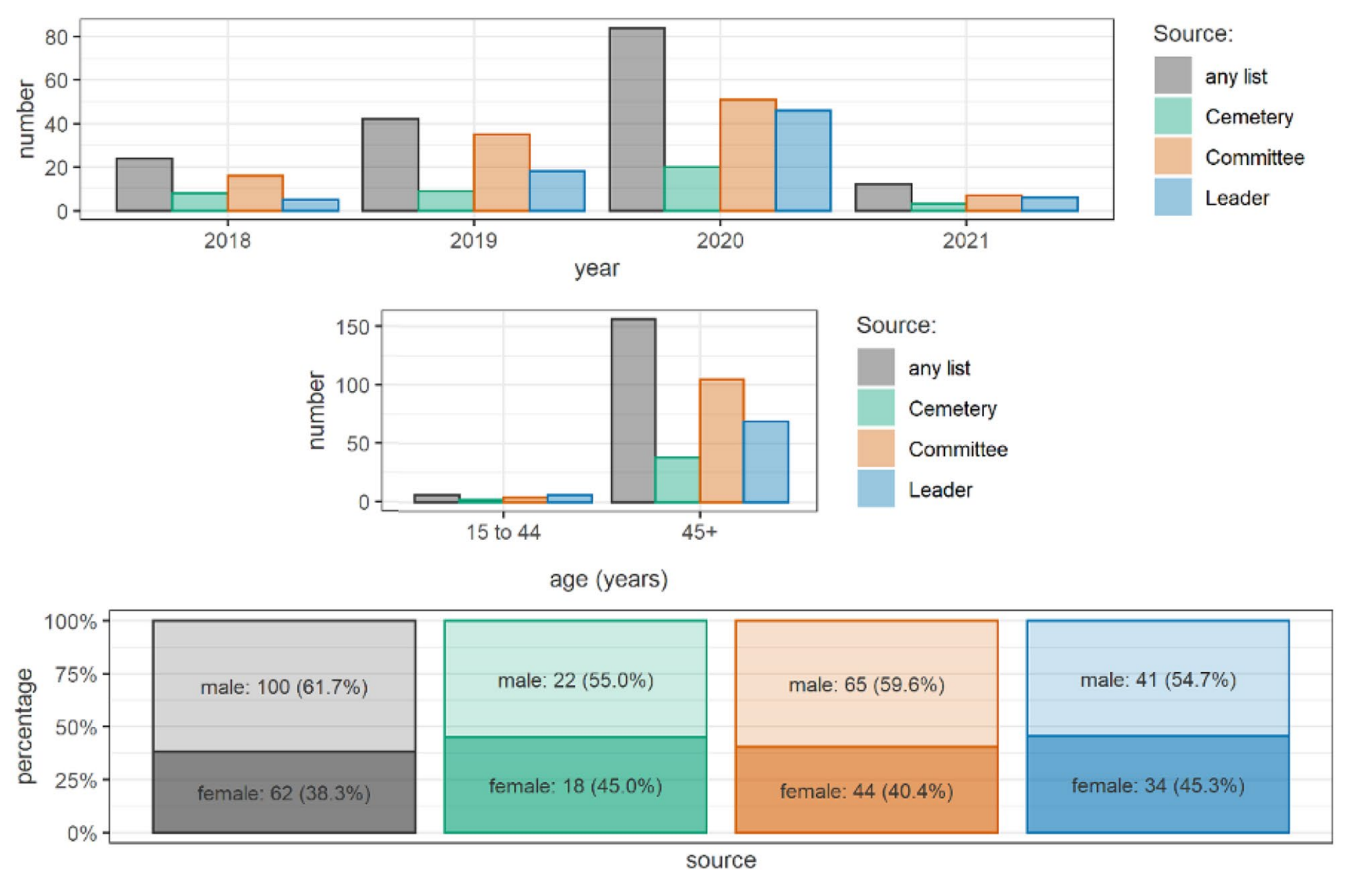
Age and gender distribution of reported decedents, Abu Haraz village - East Gezira locality

In the pre-pandemic period (January 2018 – March 2020), there was an estimated total of 109 (95% CI 86 to 179) deaths, while in the period after the start of the pandemic (March 2020- February 2021), the estimation yielded some 256 (95% CI 117 to 1326) deaths. Table 3 shows the daily death estimates for the pre-pandemic (January 2018 – March 2020) vs. the period after the start of the pandemic (March 2020- February 2021), assuming constant population denominators in each site. The death rate per day was 5.5 times higher in the period after the start of the pandemic compared to the pre-pandemic period or 447% above the pre-pandemic baseline. Of all the lists, the committee list had the highest sensitivity to detecting mortality, at 29.9% (95% CI 7.2–53.7%). Table 4 shows the sensitivity for each list. Most of the decedents were males older than 44, and the highest number of reported deaths in the pre-pandemic period was in 2019 (Fig. 2).

**Table 2:** Abu Haraz village - East Gezira locality, estimated number of deaths based on model averaging

Model	Deaths Outside Any List (95%CI)	Deaths Outside Any List (95%CI) 15/01/2018 to 12/03/2020	Deaths Outside Any List (95%CI) 13/03/2020 to 15/02/2021	AIC	Posterior Probability
No Interactions	63 (34 to 115)	28 (14 to 54)	35 (20 to 61)	873.78	0.156
Cemetery X Committee, Cemetery X Community leaders, Committee X Community leaders	Model did not fit	Model did not fit	Model did not fit	screened out	
Cemetery X Committee, Cemetery X Community leaders	61 (28 to 135)	18 (7 to 45)	43 (21 to 90)	877.56	0.023
Cemetery X Committee, Committee X Community leaders	141 (26 to 908)	72 (8 to 636)	69 (18 to 271)	877.66	0.022
Cemetery X Community leaders, Committee X Community leaders	310 (47 to 2311)	40 (14 to 114)	270 (33 to 2197)	871.28	0.543
Cemetery X Committee	56 (27 to 118)	22 (9 to 54)	34 (18 to 64)	877.11	0.029
Cemetery X Community leaders	66 (34 to 128)	24 (12 to 49)	42 (22 to 79)	874.67	0.1
Committee X Community leaders	99 (40 to 249)	48 (19 to 122)	52 (21 to 127)	874.19	0.127
Overall weighted estimate of deaths	203 (41 to 1344)	37 (14 to 107)	166 (27 to 1236)		

**Table 3:** Estimated deaths per day, based on the number of days in pre- and period after the start of the pandemic

	Estimated Total Deaths			Estimated Deaths Per Day		
Site	overall	January 2018 – March 2020	March 2020- February 2021	overall	January 2018 – March 2020	March 2020- February 2021
Abu Haraz village - East Gezira locality	365 (95%CI 203 to 1506)	109 (95% CI 86 to 179)	256 (95% CI 117 to 1326)	0.32	0.14	0.74
Hai al Quba neighbourhood- El Obeid City	188 (95%CI 114 to 498)	111 (95% CI 69 to 248)	78 (95% CI 46 to 249)	0.16	0.14	0.23

**Table 4:** Lists sensitivity

List	Number of Deaths	Sensitivity (95%CI)
Abu Haraz village - East Gezira locality		
Leader	75	20.5% (5.0–36.9%)
Cemetery	40	11.0% (2.7–19.7%)
Committee	109	29.9% (7.2–53.7%)
All Lists	162	44.4% (10.7–79.8%)
Hai al Quba neighbourhood- El Obeid City		
Women	22	11.7% (4.4–19.3%)
Committee	53	28.2% (10.6–46.5%)
Zakat	38	20.2% (7.6–33.3%)
All Lists	85	45.2% (17–74.6%)

### Hai Al Quba - El obeid city

Overall, the three lists contained 105 deaths (13 before 2018 and 10 deaths < 15 years, of whom three were in 2017 and thus all excluded), resulting in three lists with unique records for 85 decedents, with little overlap among them (See Additional Fig. 2). Table 5 shows the eight candidate models fitted to the three-list data up to 28 January 2021 and the resulting model averages. Overall, the averaging suggests some 103 (95%CI 29 to 413) deaths were not captured on any list, yielding a total estimated death toll of 188 (95%CI 114 to 498) up to 28 January 2021.

**Fig. 3:**
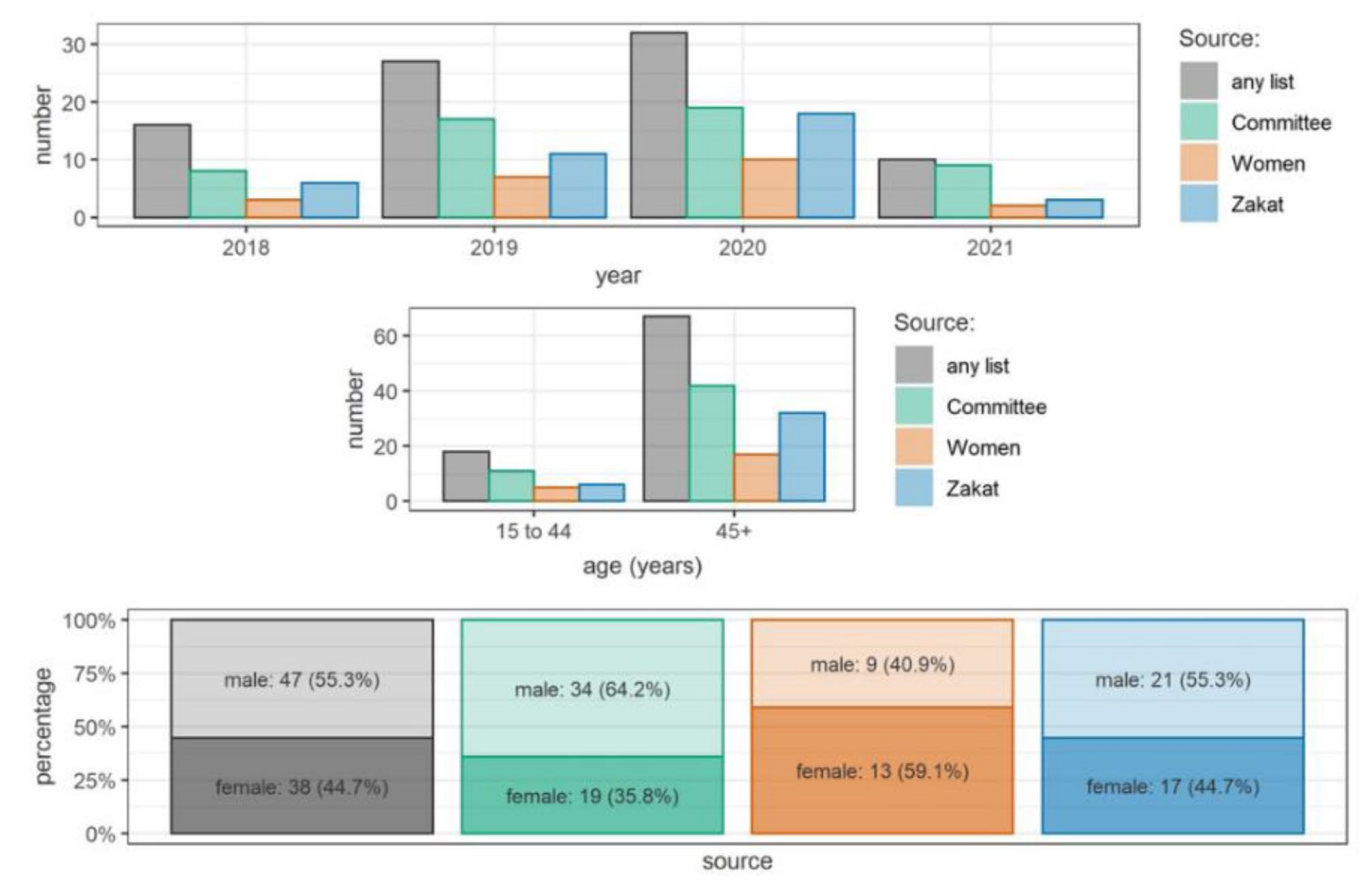
Age and gender distribution of reported decedents, Hai al Quba neighbourhood- El Obeid City

The estimation in the pre-pandemic period resulted in 111 (95% CI 69 to 248) total estimated deaths, while in the period after the start of the pandemic, 78 (95% CI 46 to 249) total deaths were estimated. The death rate per day was 1.6 times higher in the period after the start of the pandemic compared to the pre-pandemic period or 39% above pre-pandemic levels (see Table 3). Taken together, the three lists captured some 45.2% (95% CI 17–74.6%) of all deaths (Table 4). More than half of the reported decedents were males aged older than 44 years, and the highest recall pre-pandemic was in 2019 (Fig. 3).

**Table 5:** Hai al Quba - El Obeid City, estimated number of deaths based on model averaging

Model	Deaths Outside Any List (95%CI)	Deaths Outside Any List (95%CI) 15/02/2018 to 12/03/2020	Deaths Outside Any List (95%CI) 13/03/2020 to 28/01/2021	AIC	Posterior Probability
No Interactions	61 (26 to 143)	51 (22 to 119)	10 (4 to 24)	462.87	0.055
Committee X Women, Committee X Zakat, Women X Zakat	Model did not fit	Model did not fit	Model did not fit	screened out	
Committee X Women, Committee X Zakat	Model did not fit	Model did not fit	Model did not fit		
Committee X Women, Women X Zakat	127 (32 to 525)	71 (22 to 234)	56 (11 to 290)	458.22	0.565
Committee X Zakat, Women X Zakat	105 (16 to 787)	57 (11 to 300)	48 (5 to 486)	462.52	0.066
Committee X Women	81 (29 to 227)	67 (24 to 187)	14 (5 to 40)	464.6	0.023
Committee X Zakat	65 (18 to 243)	57 (15 to 210)	8 (2 to 33)	466.7	0.008
Women X Zakat	67 (26 to 174)	50 (20 to 126)	18 (7 to 47)	459.61	0.282
The overall weighted estimate of deaths	103 (29 to 413)	63 (21 to 200)	41 (9 to 212)		

## Discussion

Both communities studied, exemplifying rural and urban settings in Sudan, appeared to experience considerable excess mortality coinciding with the COVID-19 pandemic. Although we did not distinguish between COVID-19 and non-COVID-19 attributable deaths in our data collection and analysis, we are unaware of any other nonseasonal health crises that would explain the excess mortality in either location during the pandemic. It is, therefore, plausible that the observed excess mortality is mainly due to the COVID-19 pandemic (directly or indirectly). This is important to note as official figures for the entire states show very low numbers of reported COVID-19 attributable deaths.

It is reasonable to assume that COVID-19 infection and related deaths would be higher in an urban community like Hai al Quba compared to Abu Haraz village, a rural area [13]. However, our results indicate that in East Gezira, death between March 2020 and February 2021 was 447% above normal (non-crisis death), while in Hai al Quba, death was 39% above normal (non-crisis death); this could be due to lower adherence to COVID-19 preventative strategies [14, 15].

Our results coincide with previously published global excess mortality estimations between Jan 2020- Dec 2021, which were suggested to be about 3 times higher than the globally reported number of COVID-19-related deaths [16, 17]. While in high-income countries in Europe, high excess mortality rates were robustly measured during the pandemic, more fragmentary evidence also shows that low and middle-income countries in the Middle East and Africa were not, as popularly thought, spared the mortality impact of COVID-19 [16, 18]. Modelling analyses of Lebanon, Tunisia, Libya, Namibia and other low-middle income countries in the Middle East and Africa suggest high rates of excess mortality [4, 16, 19]. In Yemen, which has a similar epidemiological profile and health system to Sudan, a study reported a 230% weekly increase in excess burials in Aden during the pandemic up to September 2020 [6].

Mathematical modelling previously estimated that only 2% of COVID-related deaths were reported in Khartoum, Sudan [4] and under-ascertainment on a serious scale has also been previously reported in Syria [5], Peru [20], Brazil [21] and several settings globally [22, 23]. Lack of access to health care services, overburdened health services, lack of critical medicines, restricted food access and pandemic control measures, in addition to disruptions in humanitarian services, could all have contributed considerably to increased non-COVID-19 attributable mortality [24–26].

### Limitations

The cause of death could not be collected or verified from community key informants, and deaths occurred largely out of hospitals. Therefore, estimated all-cause mortality may be reflective not only of COVID-19 but also of seasonal epidemics, flooding, and food insecurity. However, given that these non-COVID causes of death were present pre-pandemic, we believe that the excess mortality observed, compared to baseline, was mainly attributable to COVID-19.

Capture re-capture analysis is increasingly being used to estimate excess mortality. However, it is limited by potential bias when reporting sources (key informant categories) and the resultant decedent lists are not independent of each other [27]; in three-list analysis, this bias is mitigated by introducing potential interaction terms and averaging resultant alternative models. There was little to moderate overlap between the different lists, and it is possible that key informants did not have sufficient on-the-ground coverage of the study locations to accurately detect and report every death in the study locations. Applying the study in a smaller and more refined geographical area may address this. The capture-recapture method is heavily dependent on the accuracy of record linkage. In our study, record linkage was completed by going through the deaths one by one, and any instance of ambiguous records, e.g., similar names or different reported ages of deaths, was decided through discussions with the community-based data collectors, who often knew decedent families personally. This may have minimised errors in linkage, which would tend to artificially reduce or increase list overlap and thus result in an over- or underestimation of mortality.

In the analysis, age was limited to 15 years and above as no child deaths were reported in Abu Haraz village. Only 10 child deaths were reported in Hai al Quba during the entire recall period (2018–2021). This could be because, generally, individuals tend to remember adult deaths. This might have been avoided if a separate question about the deaths of children in the community had been introduced to the questionnaire. In both locations, the lists only had 12–13 recalled deaths in 2017, which is why the analysis was restricted to deaths from 2018 onwards, possibly indicating diminishing recall ability over time and memory failure [28]. The 12% participants’ refusal rate and recall bias may have reduced the number of records and produced a sparse dataset without ample unique identifiers to allow cross-linkage.

## Conclusion

Despite facing difficulties in accurately ascertaining the causes of death due to restricted data sources and occurrences of deaths outside of medical facilities, our study underscores problems with the capture-recapture technique, emphasizing the necessity for improved delineation of geographical regions and precise record linkage. Furthermore, it highlights the underreporting of child fatalities and the diminishing ability to recall events over time.

Our study indicates that using a community-led capture-recapture methodology to generate and cross-analyse decedents lists from different community key informants to measure excess mortality is a feasible approach in Sudan and similar settings. Deploying similar community-led estimation methodologies should be considered wherever crises and weak health infrastructure prevent an accurate and timely real-time understanding of epidemics’ mortality impact in real-time [8, 29].

### Electronic supplementary material

Below is the link to the electronic supplementary material.

Supplementary Material 1

Supplementary Material 2

## Data Availability

All statistical code, and a dummy dataset that can be used to implement capture-recapture methods, are published at https://github.com/francescochecchi/mortality_capture_recapture_analysis.git. The datasets generated and analysed during the current study are available from the corresponding author upon reasonable request.
