# Supplementary material for: Population mortality before and during the COVID-19 epidemic in two Sudanese settings: a key informant study
Source: BMC Public Health. 2024 Mar 5;24:701. doi: 10.1186/s12889-023-17298-9 (PMC10916139; doi:10.1186/s12889-023-17298-9)

Supplementary Figure 1. Abu Haraz village - East Gezira locality overlap among the three lists of deceased people used for this analysis


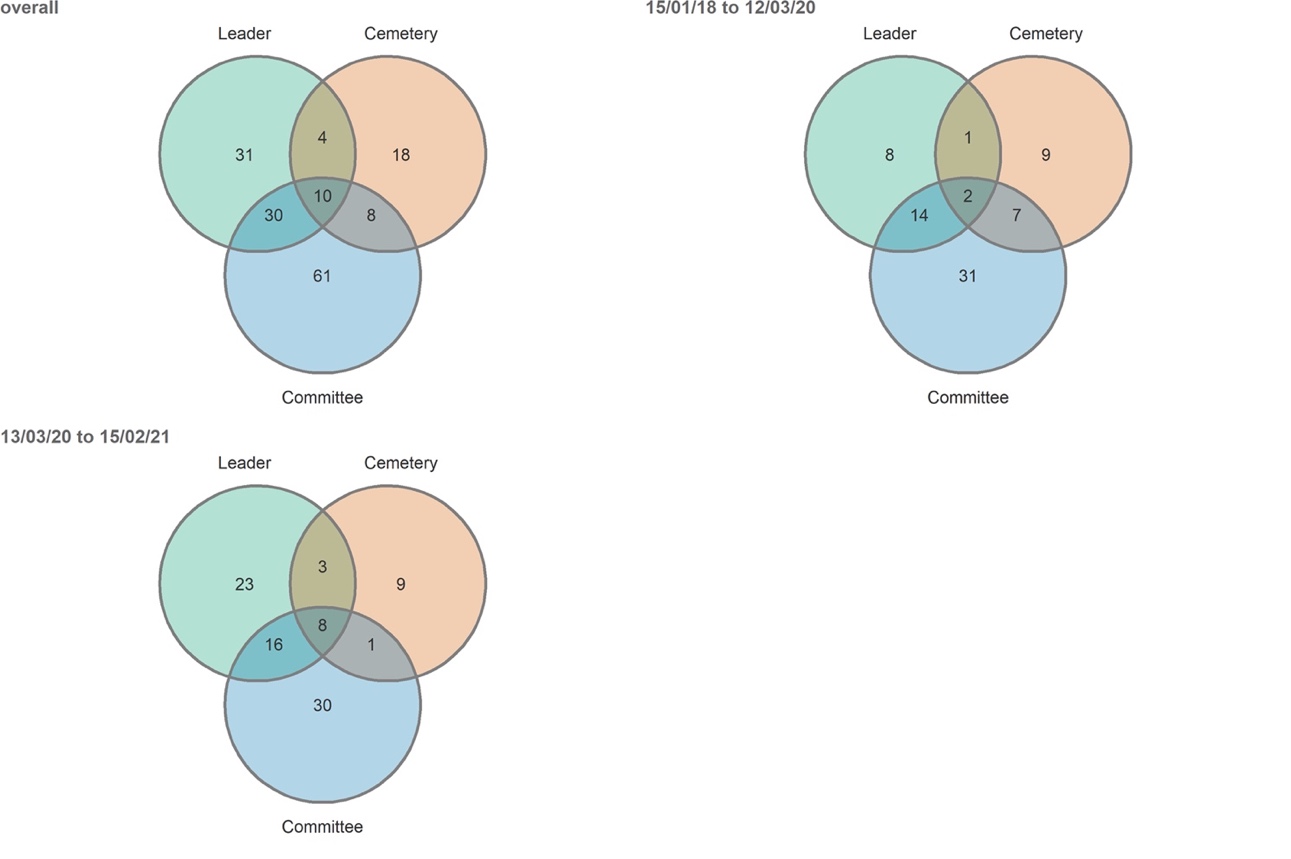

Supplement: Supplementary file 1 — Supplementary Material 1 [file 12889_2023_17298_MOESM1_ESM.docx]
